# Bacterial‐induced calcium oscillations are common to nitrogen‐fixing associations of nodulating legumes and non‐legumes

**DOI:** 10.1111/nph.13464

**Published:** 2015-05-26

**Authors:** Emma Granqvist, Jongho Sun, Rik Op den Camp, Petar Pujic, Lionel Hill, Philippe Normand, Richard J. Morris, J. Allan Downie, Rene Geurts, Giles E. D. Oldroyd

**Affiliations:** ^1^ John Innes CentreNorwich Research ParkNorwichNR4 7UHUK; ^2^ Department of Plant ScienceLaboratory of Molecular BiologyWageningen UniversityDroevendaalsesteeg 16708PBWageningenthe Netherlands; ^3^ Ecologie MicrobienneCentre National de la Recherche Scientifique UMR 5557Université Lyon IUniversité LyonVilleurbanneFrance

**Keywords:** actinorhizal, calcium oscillations, *Frankia*, legumes, nitrogen‐fixing clade, nodulation, *Parasponia*, symbiotic signalling

## Abstract

- Plants that form root‐nodule symbioses are within a monophyletic ‘nitrogen‐fixing’ clade and associated signalling processes are shared with the arbuscular mycorrhizal symbiosis. Central to symbiotic signalling are nuclear‐associated oscillations in calcium ions (Ca^2+^), occurring in the root hairs of several legume species in response to the rhizobial Nod factor signal.
- In this study we expanded the species analysed for activation of Ca^2+^ oscillations, including non‐leguminous species within the nitrogen‐fixing clade.
- We showed that Ca^2+^ oscillations are a common feature of legumes in their association with rhizobia, while *Cercis*, a non‐nodulating legume, does not show Ca^2+^ oscillations in response to Nod factors from *Sinorhizobium fredii* NGR234. *Parasponia andersonii*, a non‐legume that can associate with rhizobia, showed Nod factor‐induced calcium oscillations to *S. fredii* NGR234 Nod factors, but its non‐nodulating sister species, *Trema tomentosa*, did not. Also within the nitrogen‐fixing clade are actinorhizal species that associate with *Frankia* bacteria and we showed that *Alnus glutinosa* induces Ca^2+^ oscillations in root hairs in response to exudates from *Frankia alni*, but not to *S. fredii* NGR234 Nod factors.
- We conclude that the ability to mount Ca^2+^ oscillations in response to symbiotic bacteria is a common feature of nodulating species within the nitrogen‐fixing clade.

## Introduction

Plants have evolved endosymbioses with soil microorganisms that facilitate nutrient acquisition, including the association with arbuscular mycorrhizal (AM) fungi (Harrison, 2005), which evolved some 450 million yr ago and occurs in over 80% of higher plants (Remy *et al*., 1994; Redecker *et al*., 2000; Humphreys *et al*., 2010). Within the last 60 million yr, other symbiotic associations emerged, including the mutually beneficial relationship between plants and different nitrogen‐fixing soil bacteria, which are harboured in nodules on the roots. These root‐nodule symbioses are found in a number of plant families, all of which exist within the ‘nitrogen‐fixing clade’ (Soltis *et al*., 1995), and recent work reveals that a single innovation drove the emergence of nitrogen‐fixing symbioses resulting in nodulation (Werner *et al*., 2014). Nodulating plant species within the nitrogen‐fixing clade associate with two different groups of bacteria: filamentous Gram‐positive *Frankia* bacteria (Stacey *et al*., 1992) or Gram‐negative rhizobia (Sprent, 2009). Plant species from eight different families (referred to as actinorhizal) can associate with *Frankia*, but only legumes (*Fabaceae*) and *Parasponia* (*Cannabaceae*) form the rhizobial association (Trinick, 1973, 1979).

The establishment of the rhizobial and *Frankia* associations involves plant recognition of diffusible signals from the bacteria. Rhizobia produce lipochitooligosaccharides (LCOs), termed Nod factors, with specific modifications to the common LCO backbone, which are important in defining the plant host range of different rhizobial species (Dénarié *et al*., 1996). Recognition of Nod factors by legume roots involves two distinct LysM receptor kinases, NFR1 and NFR5 in *Lotus japonicus* and LYK3 and NFP in *Medicago truncatula*, which function in a heterodimeric complex (Madsen *et al*., 2003, 2011; Arrighi *et al*., 2006; Broghammer *et al*., 2012; Pietraszewska‐Bogiel *et al*., 2013). Upon perception of Nod factors, these receptors activate a symbiotic signalling pathway that includes an additional LRR‐type transmembrane receptor (SYMRK/DMI2) and nuclear‐localized components among which is a calcium and calmodulin‐dependent protein kinase (CCaMK) (Oldroyd, 2013). In legumes the genes encoding the Nod factor receptors underwent several duplication events, for example the ancestral LjNFR1a/MtLYK3 gene experienced a tandem duplication at the birth of the legume family (De Mita *et al*., 2014). This event most likely occurred before the evolution of the rhizobial symbiosis, as signatures of the duplication can be found in basal legume species that do not nodulate. Such ancestral duplication in LjNFR1a/MtLYK3 may have provided a degree of freedom to evolve specificity towards rhizobial Nod factors (Op den Camp *et al*., 2011a; Young *et al*., 2011).

The structure of the diffusible signal produced by *Frankia* bacteria has yet to be defined; however, the analysis of exudates and the genome sequences of different *Frankia* species suggests that these molecules have features that set them apart from canonical rhizobial LCOs (Normand *et al*., 2007; Kucho *et al*., 2010). Nevertheless, the *Frankia* symbiosis relies, at least in part, on the symbiosis signalling pathway, as at least two components are required in actinorhizal species for the *Frankia* association: SYMRK and CCaMK (Gherbi *et al*., 2008; Markmann *et al*., 2008; Svistoonoff *et al*., 2013). This suggests that even though nodulation evolved independently on multiple occasions, the utilization of symbiosis signalling for recognition of nitrogen‐fixing bacteria is a common feature within the nitrogen‐fixing clade (Markmann & Parniske, 2009).

Oscillations in the calcium ion concentration (Ca^2+^) associated with the nucleus are at the core of the symbiosis signalling pathway (Oldroyd, 2013). Nod factor‐induced Ca^2+^ oscillations have been observed in a number of different legume species, including *M. truncatula* (Wais *et al*., 2000), *Pisum sativum* (Walker *et al*., 2000), *Phaseolus vulgaris* (Cardenas *et al*., 1999) and *L. japonicus* (Harris *et al*., 2003). These species are restricted to Papilionoideae legumes. Non‐nodulating legumes and nodulating non‐legumes have not been analysed. In this study we asked whether Ca^2+^ oscillations are a common feature of bacterial recognition within the nitrogen‐fixing clade. We analysed calcium responses in a variety of legumes that provided representative species across the diversity of legumes and analysed calcium responses in the nodulating non‐legumes *Parasponia andersonii* and *Alnus glutinosa*. Our work reveals that Ca^2+^ oscillations are a conserved feature of bacterial recognition in all nodulating species analysed.

## Materials and Methods

### Plant growth

Unless stated otherwise, all plants were grown in a controlled environment at 20°C, under 16 : 8 h, light : dark conditions. The relative humidity was 32% and the light intensity was 300 μmol m^−2^ s^−1^. When aminoethoxyvinylglycine (AVG) was added to the medium, it was always to a concentration of 0.1 μM. Seeds were scarified either with sandpaper or with H_2_SO_4_ to help germination and subsequently left in bleach for 2–15 min, depending on the species. After washing with dH_2_O approximately seven times, the seeds were allowed to imbibe while rotating for 4 h at room temperature and then placed on distilled water agar (DWA) media in a Petri dish upside down overnight at room temperature. When the seeds had germinated, they were grown vertically on plates of buffered nodulation medium (BNM) with added AVG. The exception was *Cercis siliquastrum*, seeds of which were cut open after imbibing, the embryos removed and transferred to woody plant media (WPM) plates. When the plants were large enough to handle, they were moved to EKM (Becking, 1983) plates with added AVG and grown between filter papers. For transformation with NupYC2.1, *Parasponia andersonii* (clone WU1) and *Trema tomentosa* (clone WU10) were clonally propagated and transformed using *Agrobacterium rhizogenes*, as described previously (Op den Camp *et al*., 2011b; Cao *et al*., 2012).

### NGR234 Nod factor preparation

Strain ANU2811 (Chen *et al*., 1985), an exopolysaccharide defective derivative of *Sinorhizobium* sp. NGR234, was grown for 2 d in 2 l of Y medium with added Apigenin (1 μM). The culture was centrifuged at 5°C, 6000 ***g***, for 60 min. The supernatant was then passed through a C18 : 1 reverse‐phase column (Sep Pak, Waters, Elstree, UK) and eluted with 4 ml of MeOH.

The Nod factor activity was tested using root hair deformation in *Vicia hirsuta*. For this, *V. hirsuta* seeds were germinated and roots were allowed to grow to *c*. 1 cm long. Cover slides were attached to sterile microscope glass slides using silicon grease and seedlings placed between the cover slip and the slide, with the root in Fahraeus plant (FP) medium. Dilutions of the NGR234 Nod factor preparation, plus positive and negative controls, were added to the FP medium. These were left at room temperature in the dark overnight and then assessed for root hair deformation.

The NGR234 Nod factors were analysed by high‐pressure liquid chromatography coupled to a Shimadzu IT‐ToF mass spectrometer. Nod factors were separated on a 50 × 2.1 mm 2.7 μ Kinetex XB‐C18 column (Phenomenex, Macclesfield, UK) using the following gradient of acetonitrile (B) vs 0.1% formic acid in water (A), run at 600 μl min^−1^ and 40°C: 0 min, 5% B; 11.67 min, 95% B; 14.58 min, 95% B; 15.17 min, 5% B; 18.08 min, 5% B. Products were detected by positive and negative mode electrospray, with automatic data‐dependent collection of MS2 data at an isolation width of *m*/*z* 3.0, 50% collision energy and 50% collision gas. Spray chamber conditions were 250°C curved desorbation line, 300°C heat block, 1.5 l min^−1^ nebulizer gas, and drying gas ‘on’. The instrument was calibrated using sodium trifluoroacetate according to the manufacturer's instructions before use. In the negative mode, we observed *m*/*z* peaks corresponding to NodNGR‐V (Carb_2_, NMe, C18 : 1MeFuc, S) and derivatives of that with one or no carbamoyl groups (masses 1594.69, 1551.69 and 1508.68, respectively) in a ratio of *c*. 11 : 7 : 2 based on peak abundance. There were low numbers (*c*. 1/20th) of molecules with masses two units higher, presumably corresponding to the C18 : 0 variants. There were also masses of 1568.68, 1525.67 and 1482.66 which would correspond with the C16 : 0 derivatives and these were present at *c*. 25% of the level of the C18 : 1 compounds. In the positive mode, we observed masses corresponding to the non‐sulphated Nod factors NodNGR‐V (Carb_1_ or Carb_2_, NMe, C18 : 1 MeFuc) and NGR‐V (Carb_1_ or Carb_2_, NMe, C18 : 1, MeFucAc). All these correspond to the Nod‐factors described previously (Price *et al*., 1992). Using tandem MS we observed many of the expected fragments as described previously (Price *et al*., 1992). There were also some ions observed in the negative mode that we were unable to attribute and we did not see the corresponding peaks in the positive mode. We conclude that the mixture of Nod factors that we used is at least as complex as described previously (Price *et al*., 1992). To quantify the Nod‐factors present, we mixed a known concentration of *Sinorhizobium meliloti* Nod factor with the NGR234 Nod‐factor preparation and estimated the concentration of NodNGR‐V (Carb_2_, NMe, C18 : 1, MeFuc S) relative to SmIV (Ac,S, C16 : 2) by estimating relative abundance using negative mode MS; this quantification is based on the assumption that both sulphated Nod‐factors will ionize equally.

### *Frankia* factor preparation

*Frankia alni* strain ACN14a (Normand & Lalonde, 1982) was grown under nitrogen‐replete conditions as described previously (Alloisio *et al*., 2010). Mycelia from this culture were harvested by centrifugation at 700 ***g*** for 5 min. The supernatant was discarded and the bacteria (*c*. 50 mg) were resuspended in 1 ml of sterile distilled water. After vortexing, the suspension was sonicated for 30 s and centrifugated at 13 000 ***g*** for 5 min. The supernatant was collected and filtered using a 0.20 μm syringe filter (Sartorious, Epsom, UK). Supernatant (300 μl) was added to a bath of *c*. 1 ml of BNM containing the *Alnus glutinosa* seedling with microinjected cells for analysis of Ca^2+^.

### Calcium imaging

#### Cameleon NupYC2.1

The nuclear‐targeted cameleon NupYC2.1 was used for analysing Ca^2+^ responses in *P. andersonii* and *T*. *tomentosa*. The plants were inspected on a florescent stereomicroscope and roots expressing a strong signal were selected. These were removed and transferred to a microscope slide with liquid BNM media. Fluorescence resonance energy transfer (FRET) measurements were taken using an epifluorescence microscope. Cyan fluorescent protein (CFP) was excited by 437 nm with an 11 nm bandpass using an Optoscan Monochromator (Cairn Research, Faversham, Kent, UK). An image splitter (Optosplit, Cairn Research) allowed analysis of the same image through 485 and 535 nm filters to measure CFP and yellow fluorescent protein (YFP) emissions.

#### Microinjection

This protocol follows Wais *et al*. (2000). The plants were prepared as described and placed in BNM media on coverslips. The dyes used were Oregon Green, which responds to changes in Ca^2+^, and Texas Red, which is non‐responsive to Ca^2+^ and allows pseudoratiometric imaging. Both dyes were fused to a 10 kD Dextran molecule to limit free diffusion. Glass needles were prepared using an electrode puller (model 773; Campden Instruments Ltd, Loughborough, UK) from borosilicate glass capillaries (1B120F‐4; World Precision Instruments Inc., Sarasota, FL, USA). The needle was back‐loaded with 0.5 μl of the dye solution with a Microloader pipette tip. The needle was then back‐filled with *c*. 10 μl 1 M KCl. Once injected, the dye was forced from the needle into the plant cell using iontophoresis. After injection, only those cells that showed strong cytoplasmic streaming were used in the experiments. Imaging was performed using a Nikon TE2000U inverted microscope (Kingston Upon Thames, UK) and a Hamamatsu Photonics digital CC camera. Images were collected every 5 s with a 1 s exposure.

## Results

### Nod factor induced calcium oscillations in a range of legume species

**Figure 1:**
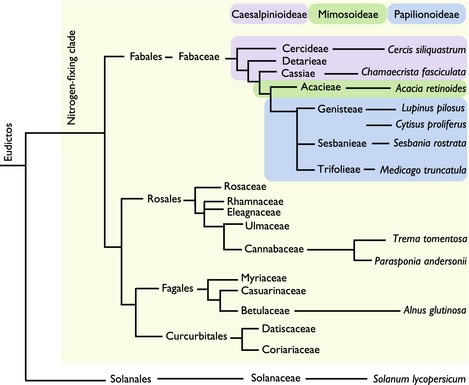
An overview of the phylogenetic relationships of plant species included in this study. The three subfamilies of the *Fabaceae* are indicated with coloured boxes. The tree is unscaled and *Solanum lycopersicum* (within the *Asterids*) is included as an outgroup.

In order to address whether Ca^2+^ oscillations are a common feature of rhizobial entry into legumes, we chose a number of legumes that represent the diversity present in this family (Fig. 1). We used *Chamaecrista fasciculata* as a representative nodulating species within the Caesalpinoideae (Naisbitt *et al*., 1992; Singer *et al*., 2009) and *C. siliquastrum* was investigated, as a representative basal, non‐nodulating legume. As a representative of the Mimosoideae subfamily, we chose *Acacia retinoides*. Within the Papilionoideae, we chose species with distinctive bacterial infection modes: *Cytisus proliferus*, which initiates root hair entry, but this aborts to be replaced by intercellular invasion (Vega‐Hernandez *et al*., 2001); and *Lupinus pilosus*, which shows intercellular invasion between epidermal cells (González‐Sama *et al*., 2004). The selection of these species was also influenced by their rhizobial symbiont: all can be colonized by the broad host range species *Sinorhizobium fredii* NGR234 (hitherto referred to as NGR234) and this allowed ease of Nod factor production and comparability across the different species (Pueppke & Broughton, 1999).

A crude isolation of NGR234 Nod factor was undertaken using published methods (Price *et al*., 1992) and the activity was validated in a root hair deformation assay on *V. hirsuta*, which is known to perceive a variety of Nod factors (Schultze *et al*., 1992). In this assay the NGR234 Nod factor preparation showed strong root hair deformation activity in a 1 : 100 000 dilution (Supporting Information Fig. S1: 10 μl of a 1 : 1000 dilution of the NGR234 NF stock in 1 ml of buffer) and therefore we used this dilution throughout the analyses of Ca^2+^ oscillations. Analysis of this Nod factor isolation revealed that this is equivalent to 0.7 nM of NodNGR‐V (Carb_2_, NMe, C18 : 1MeFuc, S), the pentameric Nod factor carrying N‐linked C18 : 1 acyl and methyl groups, two carbamoyl groups on the terminal non‐reducing glucosamine and a sulphated fucose on the reducing glucosamine residue. The other identified Nod factors (lacking one or two carbamoyl groups and with different acyl groups; see the Materials and Methods section) added up to a similar concentration, such that the total concentration of the identified Nod factors applied throughout this work was *c*. 1.5 nM.

**Figure 2:**
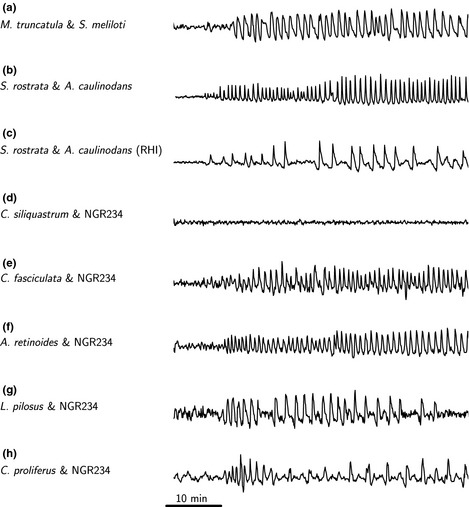
Representative traces showing Nod factor‐induced Ca^2+^ oscillations in a variety of legume species. Each panel shows a representative root hair Ca^2+^ trace from the following species: (a) *Medicago truncatula* treated with *Sinorhizobium meliloti* Nod factor; (b) *Sebania rostrata* grown under conditions that promote crack entry and treated with *Azorhizobium caulinodans* Nod factor (Capoen *et al*., 2009); (c) *S. rostrata* grown under conditions that promote root hair invasion and treated with *A. caulinodans* Nod factor (Capoen *et al*., 2009); (d) *Cercis siliquastrum* treated with NGR234 Nod factor; (e) *Chamaecrista fasciculata* treated with NGR234 Nod factor; (f) *Acacia retinoides* treated with NGR234 Nod factor; (g) *Lupinus pilosus* treated with NGR234 Nod factor; (h) *Cytisus proliferus* treated with NGR234 Nod factor.

**Figure 3:**
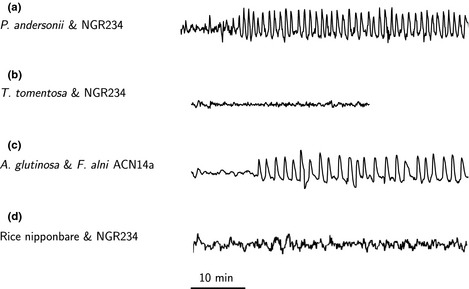
Representative traces showing bacterial‐induced Ca^2+^ oscillations in non‐legumes. Each panel shows a representative root hair Ca^2+^ trace from the following species: (a) *Parasponia andersonii* treated with NGR234 Nod factor; (b) *Trema tomentosa* treated with NGR234 Nod factor; (c) *Alnus glutinosa* treated with exudates from *Frankia alni* ACN14a; (d) rice nipponbare treated with NGR234 Nod factor.

The analysis of calcium responses on these diverse species is rather limited, as no established transformation procedures exist for most of these species and therefore we are restricted to microinjection of calcium responsive dyes. Such an approach is limited to the analysis of young growing root hair cells, as other cell types are intransigent to microinjection. We microinjected growing root hairs on the primary roots of *C. fasciculata*,* A. retinoides*,* C. proliferus*,* L. pilosus* and *C. siliquastrum* with a mixture of the Ca^2+^‐responsive dye Oregon Green and the non‐responsive dye Texas Red to allow pseudo‐ratiometric analysis of Ca^2+^ oscillations. *Chamaecrista fasciculata*,* A. retinoides*,* C. proliferus* and *L. pilosus* all responded to NGR234 Nod factor with activation of Ca^2+^ oscillations (Fig. 2; Table 1). For comparison, Ca^2+^ traces from *M. truncatula* and *Sesbania rostrata* are included in Fig. 2 (Capoen *et al*., 2009). Similar to Nod factor‐induced Ca^2+^ oscillations previously reported in *M. truncatula*,* L. japonicus* and *S. rostrata* (Ehrhardt *et al*., 1996; Wais *et al*., 2000; Miwa *et al*., 2006; Capoen *et al*., 2009), the Ca^2+^ responses we observed were activated 10–20 min after Nod factor addition, continued for over 1 h (Fig. 2) and were restricted to the nuclear region (Fig. S2). By contrast, root hairs of the non‐nodulating legume *C. siliquastrum* and the non‐legume rice (*Oryza sativa*) showed no response to NGR234 Nod factor (Figs 2d, 3d; Table 1). Taken together, this work shows that in legumes Nod factor‐induced Ca^2+^ oscillations appear to correlate with the capacity to form root nodules.

**Table 1:** Proportion of cells showing Ca^2+^ oscillations in response to NGR234 Nod factors or *Frankia* exudates (*Alnus glutinosa*), measured by either microinjection or cameleon

Plant species	Ca^2+^‐responsive cells/total cells analysed
Rice (nipponbare)	0/30
*Parasponia andersonii*	17/26
*Trema tomentosa*	0/35
*Alnus glutinosa*	5/10
*Cercis siliquastrum*	0/15
*Chamaecrista fasciculata*	13/20
*Acacia retinoides*	13/17
*Lupinus pilosus*	11/12
*Cytisus proliferus*	12/17

### Calcium oscillations in the non‐legume *P. andersonii*

Plant species in the genus *Parasponia* within the *Cannabaceae* are the only non‐legumes known to form nodules with rhizobia, and *P. andersonii* does so with NGR234 (Pueppke & Broughton, 1999). *Parasponia andersonii* is a tropical tree that can be propagated vegetatively (Op den Camp *et al*., 2011b) and plantlets from vegetative propagation were used in this study. *P. andersonii* showed Ca^2+^ oscillations in root hair cells in response to NGR234 Nod factor following microinjection (Fig. 3a; Table 1). However, microinjection of *P. andersonii* root hairs was extremely challenging and this made it difficult to gather data from a sufficient number of cells. We therefore used *A. rhizogenes* to introduce the Ca^2+^ reporter cameleon (NupYC2.1; Sieberer *et al*., 2009) into *P. andersonii* roots. When challenged with NGR234 Nod factor, these cameleon‐transformed roots revealed Ca^2+^ oscillations (Table 1). Plant species of the sister genus of *Parasponia*,* Trema*, do not nodulate and to assess Nod factor‐induced Ca^2+^ oscillations in this close relative, we transformed *T. tomentosa* roots with NupYC2.1. We observed no Ca^2+^ oscillations in *T. tomentosa* treated with NGR234 Nod factors (Fig. 3b; Table 1). Taken together, these results show that also outside the legume family, rhizobium root nodule formation is causally associated with Nod factor‐induced Ca^2+^ oscillations.

### *Frankia* factor(s) induce Ca^2+^ oscillations in the actinorhizal tree *A. glutinosa*

*Alnus glutinosa* belongs to the *Betulaceae* in the order *Fagales* and is an important colonizer in many ecosystems because of its capacity to form nitrogen‐fixing symbioses with *Frankia* bacteria (Wall, 2000). While *Frankia* produce diffusible signals, their structure(s) have yet to be defined (Cérémonie *et al*., 1999; Pawlowski *et al*., 2011). However, exudates from *Frankia* bacteria can induce root hair deformation (Pawlowski *et al*., 2011) and, therefore, we asked whether such exudates could also activate Ca^2+^ oscillations. We cultured the slow‐growing *F. alni* ACN14a for a number of months and used sonication to break up the hyphal mats. The bacteria were centrifuged and the exudates were used to treat Oregon Green/Texas Red microinjected root hairs of *A. glutinosa*. This exudate could activate Ca^2+^ oscillations in *A. glutinosa* root hair cells (Fig. 3c; Table 1) and also induced root hair deformation (Fig. S3). By contrast, *A. glutinosa* did not respond to NGR234 Nod factor, and *M. truncatula* was unable to mount calcium oscillations in response to the exudates from *F. alni* (Fig. S4). We observed a comparable delay before induction of Ca^2+^ oscillations in *A. glutinosa* and similar nuclear‐localization as previously observed in legumes (Ehrhardt *et al*., 1996).

## Discussion

All plant species that form root‐nodule symbioses are confined to the monophyletic nitrogen‐fixing clade, but most species within this clade do not nodulate (Soltis *et al*., 1995). This suggests that a genetic event within a common ancestor predisposed this group to evolve root‐nodule symbioses, followed by multiple gains and losses of the symbiosis (Werner *et al*., 2014). Here we show that bacterial activation of Ca^2+^ oscillations in root hair cells is a common feature of diverse legumes, the actinorhizal species *A. glutinosa* and the rhizobial‐associating non‐legume *P. andersonii*. This is consistent with genetic work indicating the requirement for components of the symbiosis signalling pathway in the actinorhizal species *Casuarina* (Gherbi *et al*., 2008; Markmann *et al*., 2008; Svistoonoff *et al*., 2013).

Symbiosis signalling initially evolved to support the AM association and this signalling pathway was recruited during the evolution of nodulation (Markmann & Parniske, 2009; Oldroyd, 2013). AM fungi and their exudates can activate calcium oscillations in both legumes and non‐legumes (Kosuta *et al*., 2008; Chabaud *et al*., 2011; Genre *et al*., 2013) and this is probably the result of LCOs and chitooligosaccharides (COs) present in the mycorrhizal exudates (Maillet *et al*., 2011; Genre *et al*., 2013). One possible scenario for the predisposition event is a modification to the symbiosis signalling pathway that allowed recognition of nitrogen‐fixing bacteria. We analysed two non‐nodulating species within the nitrogen‐fixing clade, *T. tomentosa* and *C. siliquastrum*, and neither were able to activate Ca^2+^ oscillations in response to NGR234 Nod factor. If these species are representative of non‐nodulating species containing the predisposition event, then we must conclude that bacterial recognition is not associated with the evolution of the predisposition to nodulate. However, caution is neccessary when concluding this based on a negative result using only NGR234 Nod factor.

*Parasponia* species are the only non‐legumes reported to form root‐nodule symbioses with rhizobia (Geurts *et al*., 2012). A very closely related sister species, *T. tomentosa*, does not nodulate and does not respond to NGR234 Nod factors. This suggests that the ability to respond to NGR234 Nod factor may have evolved very recently and could have faciliated the emergence of nodulation in *P. andersonii*. Molecular studies have revealed that the same receptor in *P. andersonii*, PaNFP, is involved in the recognition of symbiotic signalling molecules from both AM fungi and rhizobial bacteria (Op den Camp *et al*., 2011b). Possibly a minor difference within the receptor between *Trema* and *Parasponia* explains this shift in Nod factor sensitivity, or, alternatively, evolution of other LysM receptor‐like kinases could be the cause.

We have shown that a diffusible signal produced by *F. alni* can activate symbiosis signalling in *A. glutinosa* with resultant Ca^2+^ oscillations. This has analogies with LCO signalling in rhizobia; however, research in *Frankia* has implied that the diffusible signal is not a canonical LCO (Cérémonie *et al*., 1999; Normand *et al*., 2007). The nature of this signal and the receptors that are required for its recognition remain to be resolved. Our work reveals commonalities in the activation of Ca^2+^ oscillations by diffusible bacterial signalling molecules across all nodulating species tested, both legumes and non‐legumes. This highlights the importance of symbiosis signalling and related Ca^2+^ oscillations for the recognition of nitrogen‐fixing bacteria within the nitrogen‐fixing clade. The fact that mycorrhizal fungi also produce LCO and CO signalling molecules (Maillet *et al*., 2011; Genre *et al*., 2013) suggests that the capacity to perceive nitrogen‐fixing bacteria may have evolved from the perception of mycorrhizal‐associated LCO and CO signalling molecules. A full understanding of the evolutionary path that led to the perception of nitrogen‐fixing bacteria requires a detailed analysis of mycorrhizal recognition processes of plants within the nitrogen‐fixing clade. This is complicated by the fact that Myc‐LCO and CO responses appear restricted to atrichoblasts (Sun *et al*., 2015), which are intransigent to microinjection. While such an analysis of mycorrhizal‐induced calcium responses is necessary for a full understanding of the evolution of nodulation, it will require the long‐term development of transformation procedures in non‐model species.

## Supporting information

Please note: Wiley Blackwell are not responsible for the content or functionality of any supporting information supplied by the authors. Any queries (other than missing material) should be directed to the *New Phytologist* Central Office.

**Fig. S1** Root hair deformation responses of *Vicia hirsuta* to the NGR234 NF purifications.
**Fig. S2** The nuclear localization of the calcium oscillation response in a variety of legume species.
**Fig. S3** Root hair deformation in *Alnus glutinosa* following treatment with *Frankia* exudates.
**Fig. S4** Calcium traces in *Alnus glutinosa* treated with NGR234 NF and in *Medicago truncatula* treated with *Frankia* exudates.
