# Supplementary material for: Bacterial‐induced calcium oscillations are common to nitrogen‐fixing associations of nodulating legumes and non‐legumes
Source: New Phytol. 2015 May 26;207(3):551–8. doi: 10.1111/nph.13464 (PMC4736677; doi:10.1111/nph.13464)
Supplement: Supplementary file 1 — Fig. S1 Root hair deformation responses of Vicia hirsuta to the NGR234 NF purifications. Fig. S2 The nuclear localization of the calcium oscillation response in a variety of legume species. Fig. S3 Root hair deformation in Alnus glutinosa following treatment with Frankia exudates. Fig. S4 Calcium traces in Alnus glutinosa treated with NGR234 NF and in Medicago truncatula treated with Frankia exudates. [file NPH-207-551-s001.pdf]

Supporting Information Figs S1– S4

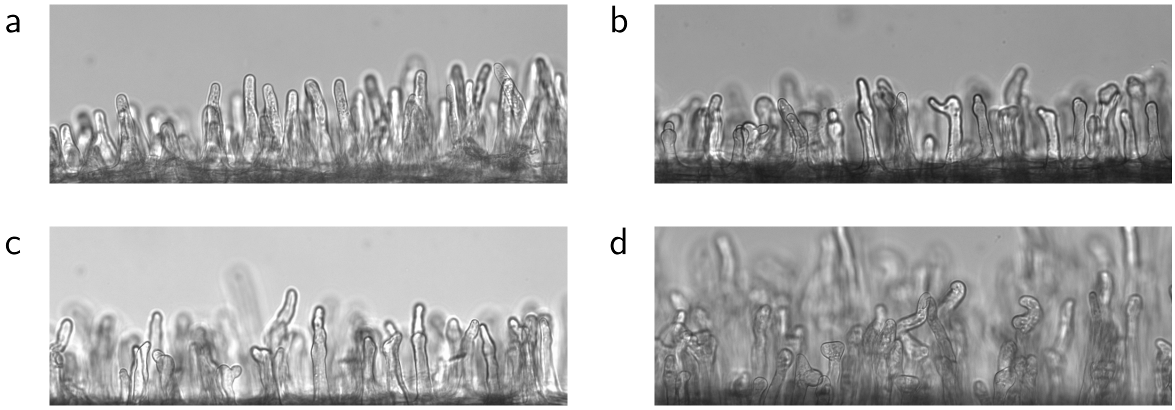

Supplementary figure 1. Root hair deformation induced by NGR23 NF treatment in *Vicia hirsuta*. **a:** Negative control. **b:** Supernatant of NGR234 liquid culture. **c:** NGR234 Nod factors, diluted 1/1000. **d:** NGR234 Nod factors diluted 1/100.

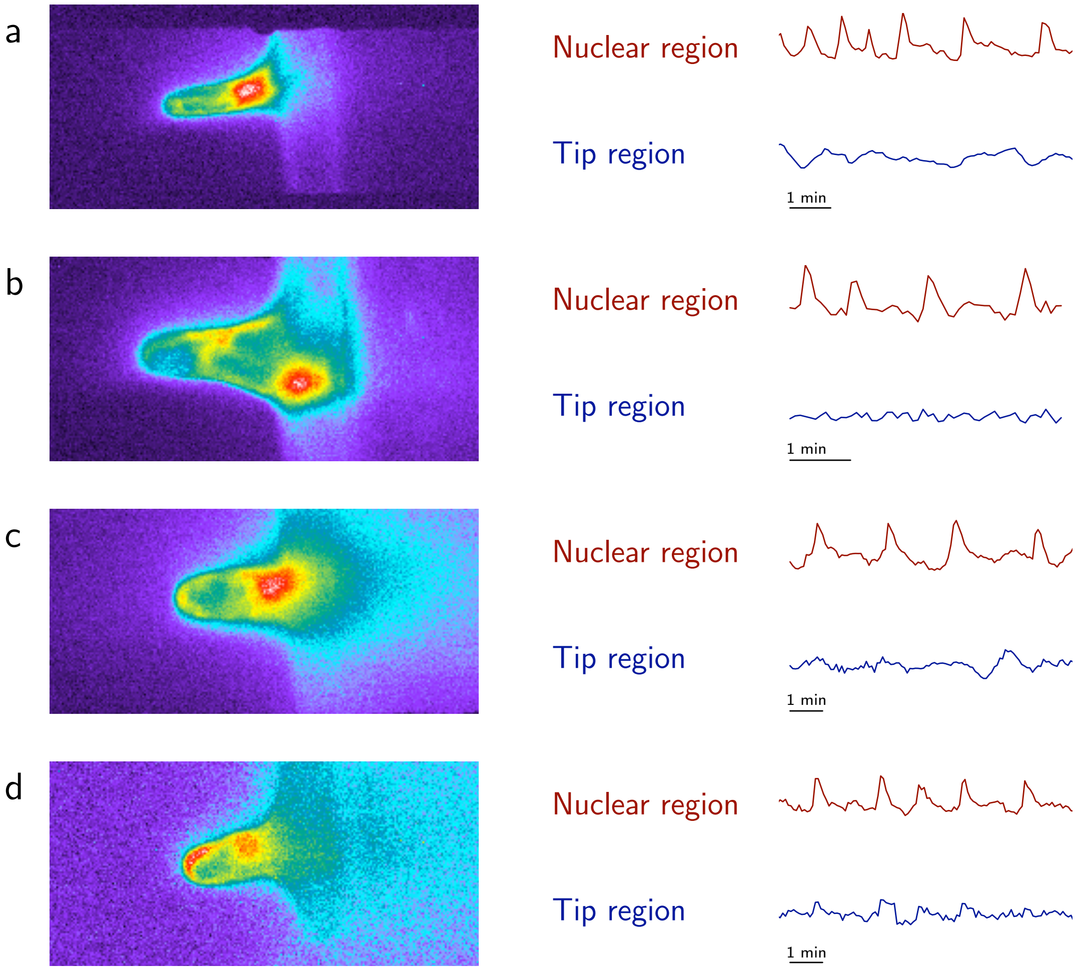

Supplementary figure 2. Nuclear localisation of Ca^{2+} spiking. Images of NF treated root hair cells of: **a:** *Lupinus pilosus*. **b:** *Chamaecrista fasciculata*. **c:** *Cytisus proliferus*. **d:** *Acacia retinoides*.

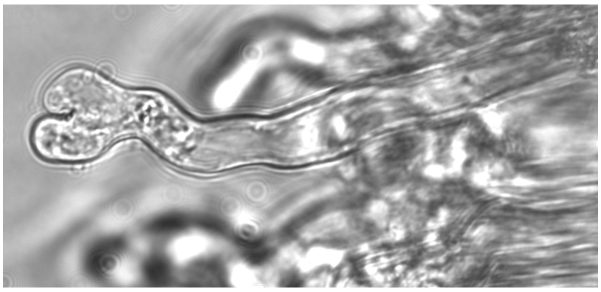

Supplementary figure 3. Root hair deformation in *A. glutinosa* after induction by Frankia exudates.

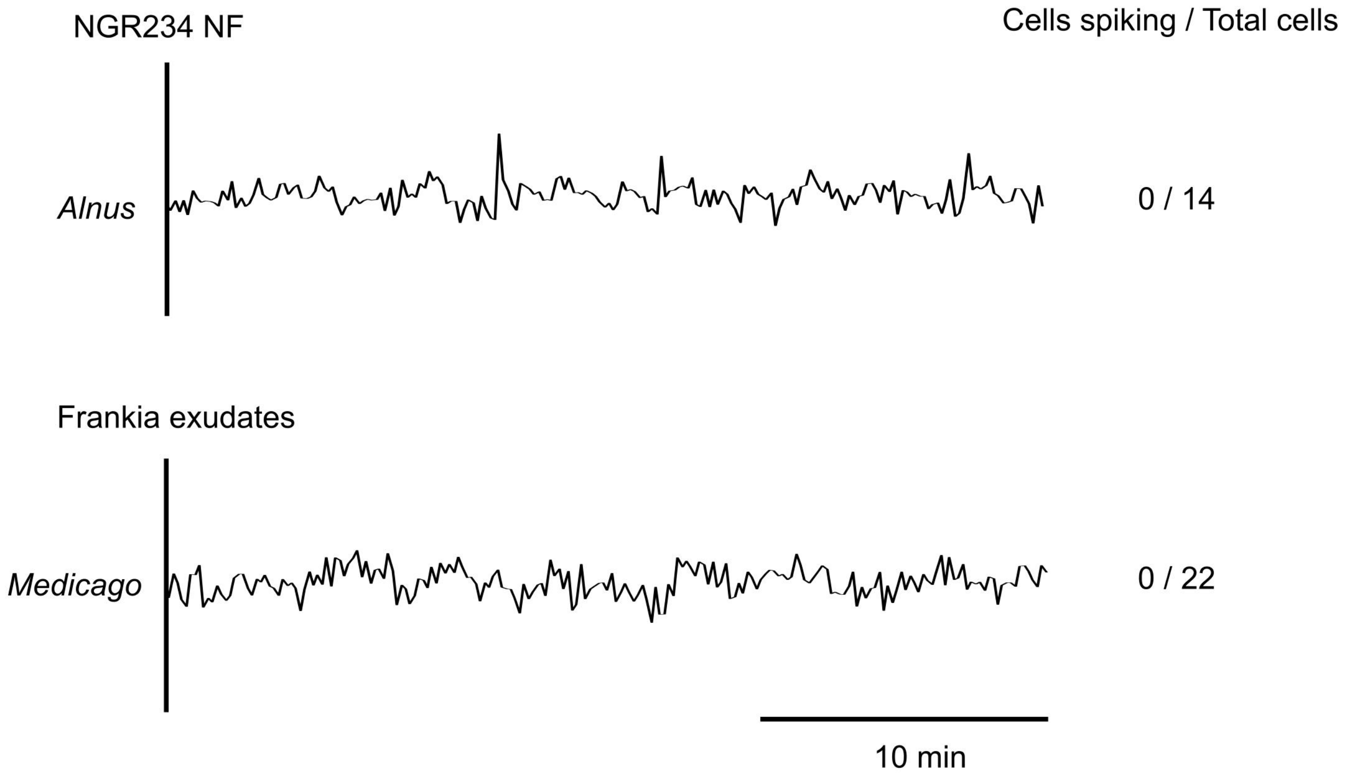

Supplementary figure 4. Ca^{2+} traces in *Alnus* treated with NGR234 NF and in *Medicago* treated with Frankia exudates. The number of responsive cells is indicated relative to the total cells analysed.
